# How parents describe the positive aspects of parenting their child who has intellectual disabilities: A systematic review and narrative synthesis

**DOI:** 10.1111/jar.12617

**Published:** 2019-05-20

**Authors:** Carole Beighton, Jane Wills

**Affiliations:** ^1^ Faculty of Health, Social Care and Education Kingston and St George's University of London London UK; ^2^ School of Health and Social Care London Southbank University London UK

**Keywords:** intellectual disabilities, learning disability, parent carers, positive aspects of parenting, systematic review

## Abstract

**Background:**

Identifying what parents describe to be positive about parenting their child who has intellectual disabilities is important for professional practice and how parents can be supported over a lifespan.

**Methods:**

Studies in which parents describe the positive aspects of parenting their child with intellectual disabilities were identified via electronic databases searches and analysed in a narrative synthesis.

**Results:**

Twenty‐two studies were included. Consistent themes emerged relating to positive change, increased personal strength, growth and development largely related to parental intrapersonal orientation. Several studies emphasized that the presence of growth or positive change does not imply the absence of distress.

**Conclusions:**

Positive aspects are not consistently defined and measured differently across studies. Consistent themes are described variously attributed to theories relating to coping, adaptation or growth following adversity; however, no single theoretical framework emerged. Factors likely to predict a parent's ability to identify positive aspects are inconclusive.

## 1 BACKGROUND

Most children with intellectual disabilities live at home with their parents. In addition, there are estimates that in England between 35% and 87% of adults with an intellectual disability also live at home and are supported by their parents, families or friends (Copeland, 2012; Hatton et al., 2014; NHS Digital, 2015), and similarly, in the USA around 78% of adults are estimated to live with family members (The Arc, 2011).

Most research on parenting a child with an intellectual disability is typically framed within a stress‐coping paradigm in which the child (or their characteristics) is “the stressor” and a the parent or family is reported as coping with a negative impact or burden from the child's impairment(s) (Hastings, Daley, Burns, & Beck, 2006; McConnell & Savage, 2015). This includes higher overall levels of poor physical, mental health and mental well‐being compared to parents of typically developing children (NHS England et al., 2016) with these poor outcomes increasing with the duration and intensity of the “caring” role (DOH, 2010; Neece & Baker, 2008). These parents also face socio‐economic disadvantage which includes increased risk of poverty, stigma and social exclusion (Emerson, 2012; Emerson & Hatton, 2014; Joseph Rowntree Foundation, 2016).

Whilst the stress that comes with parenting a child with intellectual disabilities is undeniable, this focus on a pathological model represents only one perspective and several studies report that carers can experience both stress and positive experiences simultaneously (Baker et al., 2003; Hastings et al., 2006; Patton, Ware, McPherson, Emerson, & Lennox, 2016). Although initially dismissed as denial, an attempt to alleviate their guilt or a defensive reaction (Behr, 1990; Wikler, Wasow, & Hatfield, 1983) the past twenty years has seen a growing body of literature which has recognized the positive effects and contributions that the child brings to the parents and family. However, in some studies, positive aspects are inferred only by the absence of negative affect, stress and depression (Miersschaut, Roeyers, & Warreyn, 2010; Van de Veek, Kraaij, & Garnefski, 2009).

There is no single theoretical model which addresses the idea of “positivity” in parenting a child with intellectual disabilities (Beighton & Wills, 2017; Blacher & Baker, 2007) although several different conceptual models have been proposed in general studies (Table 1). These illustrate different theoretical and ontological positions about positivity and whether it reflects a coping process (Park, 2010, 2013; Park & Folkman, 1997; Taylor, 1983) or is post‐traumatic (PTG) or adversarial growth (Hobfoll et al., 2007; Joseph & Linley, 2005; Maercker & Zoellner, 2004; Schaefer & Moos, 1998; Tedeschi & Calhoun, 1995, 2004). However, one commonality is that most of the theoretical models require individuals to use cognitive coping/processing to construct meaning from their experiences.

**Table 1:** Theoretical models of positivity

Coping models	
Coping with adverse events model/Stress related growth and thriving/meaning making model (Park, 2010, 2013)	Extends the framework of the transactional model (Lazarus & Folkman, 1984) by including meaning making in which individuals engage in positive reframing to search for a more favourable understanding of their situation. Meanings made can include changes in the way the person appraises a situation as well as changes in global meaning, such as revised identity, growth or views of the world.
Cognitive adaptation theory (Taylor, 1983)	Proposes that individuals respond to personally threatening events through a process of adjustment involving the resolution of three cognitive themes, a search for meaning, an attempt to gain mastery or control and enhancing self‐esteem. Through this process, individuals focus on the beneficial qualities of the situation and engage in active coping efforts to foster positive changes.
**Growth models**	
The Janus face model of self‐perceived growth (Maercker & Zoellner, 2004)	Considers “growth” to have both a functional, constructive side and an illusory, self‐deceptive side. The functional side is where individuals report positive changes after a stressful life event. In contrast, the illusory side is where people cope with threatening situations by positively distorting their perception of the event or themselves.
The action growth model (Hobfoll et al., 2007)	Argues that growth does not result from cognitive attempts to find meaning and re‐structure assumptive beliefs about the world but rather for growth to occur individuals must translate these cognitive benefit‐finding processes into action. As with the Janus face model, in this model there are two possible manifestations, an illusory coping side and a functional and constructive side. The illusory side (cognitive attempts to find positive benefits in adversity) might be a coping mechanism in the aftermath of extreme stress, and not really positive change.
Life crises and personal growth model (O'Leary, Sloan Alday, & Ickovics, 1998; Schaefer & Moos, 1992, 1998)	The combination of the person's environmental and coping resources prior to the event determines the outcome following a life crisis of a traumatic event. Central to this model is the assumption that coping functions as one mechanism through which personal and social resources can be used to achieve subsequent growth and positive change.
Models of posttraumatic growth(PTG) (Joseph et al., 2012; Tedeschi & Calhoun, 1996) Orgasmic valuing theory (Joseph & Linley, 2005)	Rooted in humanistic positive psychology that emphasizes features that make life worth living such as hope, wisdom, courage and perseverance. Based on the work of Janoff‐Bulman (1992), these models propose that highly traumatic or stressful events can “shatter” the individual's assumptive beliefs about their world. PTG is the transformation of the person in the aftermath of experiencing a traumatic event, which can trigger positive personal growth. The orgasmic valuing theory assumes that a person must “accommodate” the trauma by modifying their prior worldviews in a positive way in order to achieve growth.

There is no accepted definition of a positive aspect. However, it has been described as situations where the person appraises caregiving as “enhancing or enriching their life” (Kramer, 1997:219). Positive aspects have been investigated in other carer groups, for example in those caring for people with cancer (Hudson, 2004), stroke (Mackenzie & Greenwood, 2012), Alzheimer's disease (Cheng, Mak, Lau, Ng, & Lam, 2015), dementia (Lloyd, Patterson, & Muers, 2014) and developmental disabilities (Manor‐Binyamini, 2016; Strecker, Hazelwood, & Shakespeare‐Finch, 2014). Caregivers who report positive aspects have been found to have better self‐reported health, less depressive symptoms, higher caregiving competence and greater family adjustment (Basu, Hochhalter, & Stevens, 2015; Cheng, Lam, Kwok, Ng, & Fung, 2013; Pinquart & Sörensen, 2004; Trute, Benzies, Worthington, Reddon, & Moore, 2010). The parent of a child with intellectual disabilities may, however, experience positive aspects differently to other carer groups as they provide lifelong support for their child compared to the 3–15 years of exposure to physical and psychosocial demands faced, for example, by those caring for a person with Alzheimer's disease (Vitaliano, Zhang, & Scanlan, 2003). Therefore, it is important to explore whether positive aspects are reported similarly in this parent group.

## 2 AIM OF REVIEW

The aim of this review is to identify what parents describe to be positive about parenting a child with intellectual disabilities by undertaking a systematic review and narrative synthesis of primary research. A secondary aim is to identify the range of factors that may contribute to parental positive perceptions. Bringing together the evidence on such positive aspects has important implications for professional practice in how it supports parents to be able to continue caring over the lifespan.

The terms child and children are used throughout this review to represent both children and adult children.

## 3 METHODS

The decisions regarding the search strategy and inclusion criteria, study selection, data extraction, quality assessment and data synthesis are outlined following guidance from the Centre for Reviews and Dissemination (Centre for Reviews & Dissemination, 2008).

### 3.1 Search strategy

An Internet‐based bibliographic database search was initially conducted in December 2015 and updated in October 2018 using the following search engines and databases: SCOPUS (all fields); EMBASE, AHMED, MEDLINE and CINAHL Plus; Science Direct, PsycINFO, British Nursing Index (BNI), Biomed Central and Internurse. As the review was not exploring intervention studies, a PEO framework (population, exposure and outcome) was utilized (Booth, 2006) and Boolean operators were used to combine terms for parent/carer and parenting/caring for a child with an intellectual disability and synonyms used for positive aspects were identified from literature which explored positive aspects in other carer groups (as described previously). No methodological filter was applied. An example of one search string undertaken on the CINAHL Plus database is shown in Table 2.

**Table 2:** CINAHL Plus database search strategy

#	Searches/Keywords (Boolean/Phrase)	Results
S1	intellectual disabilit* OR mental retardation OR mentally handicapped OR learning disabilit* OR learning difficult* OR special needs OR developmental delay OR developmental disabilit*	23,463
S2	fathers OR mothers OR parents OR carer OR caregiver OR parent carer OR family caregiver OR families	259,315
S3	positive perceptions OR positive aspects OR positive feelings OR positive experiences OR positive impact OR gratifications OR satisfactions OR rewards OR positive reappraisal OR positive reframing OR benefit* OR posttraumatic growth OR post‐traumatic growth OR post traumatic growth OR stress‐related growth OR meaning‐focused coping OR growth following adversity OR psychological growth	225,136
S4	S1 AND S2 AND S3	654
S5	Limiters‐ Published up to 2018. English Language	614

Ancillary searching included using the Mendeley academic network, reference lists and citation trails from the included papers, and authors with published work in the field were also contacted. Grey literature and non‐peer‐reviewed studies were excluded. Studies in which positive experiences or impact of parenting were described were included if the positive aspects were described narratively and identified as findings in the abstract. The inclusion and exclusion criteria for the literature search are shown in Table 3.

**Table 3:** Inclusion and exclusion criteria

Inclusion criteria
Study has a primary focus on how parents describe the “positive aspects” of parenting a child with an intellectual disability
Describes the experiences, perceptions and views of primary carers (i.e., parents & family, not paid carers) for a child/adult child (of any age) who has a primary diagnosis of intellectual disability
The child lives at home with the parent
Published in English language
Any study design
Exclusion criteria
Positive aspects are not described by the parents (i.e., scores are reported, correlations and relationships explored or hypotheses tested)
Positive aspects only briefly mentioned
The child has autism spectrum disorder (ASD), pervasive developmental disorder or developmental delay/disabilities without a primary diagnosis of intellectual disability
The study includes a variety of children with developmental delays/developmental disabilities and results/findings are not reported separately
The child lives in residential or supported accommodation
Not published in English language

### 3.2 Screening and data extraction

Eligibility for full‐text retrieval was ascertained by screening the titles and abstracts according to the inclusion/exclusion criteria and was carried out independently by two reviewers. Three disagreements were resolved through discussion, and for one study, it was necessary to involve a third reviewer. Full texts were screened against the same criteria.

A data extraction form was developed and piloted with two studies. Data extracted included authors, study design, methodology, country of publication, the participant and child characteristics and the positive aspects identified.

### 3.3 Data analysis

Data were analysed using a textual narrative approach and thematic analysis, which is particularly suitable for identifying the main, recurrent and or most important themes (based on the review question) and provides a possible structure for new research (Lucas, Baird, Arai, Law, & Roberts, 2007). The characteristics of positive aspects reported were summarized using words and text within individual studies and then between all included studies. This data were then grouped into meaningful themes (Thomas, Harden, & Newman, 2012).

### 3.4 Quality assessment

Critical appraisal and assessment of quality of the included studies were carried out by three researchers and any queries discussed and agreement reached. Applying quality criteria rigidly is likely to exclude relevant studies simply because they fail to comply with a particular reporting regimes (Lucas et al., 2007; Joanna Briggs Institute, 2014); therefore, each article was assessed independently and no composite score for quality was made as recommended by the Cochrane methods panel (Higgins, Altman, & Sterne, 2011). A checklist (National Collaborating Centre for Methods & Tools, 2003) adapted by Greenwood, Mackenzie, Cloud, and Wilson (2008) which had been used in a systematic review on positive experiences of caregiving in stroke (Mackenzie & Greenwood, 2012) was used to interrogate the quantitative studies for their potential sources of bias (selection, measurement and confounding) and the credibility of the discussion and conclusion. Each study was considered to be of low, medium or high risk of bias, but no articles were excluded on this basis. Qualitative studies were interrogated using an adapted checklist from criteria outlined by Popay, Rogers, and Williams (1998) and adapted by Greenwood, Mackenzie, Cloud, and Wilson (2009) for use in studies of carers of stroke survivors which screened for integrity, transparency and transferability. These were considered to be “of quality” or “of a lower quality.”

## 4 RESULTS

**Figure 1:**
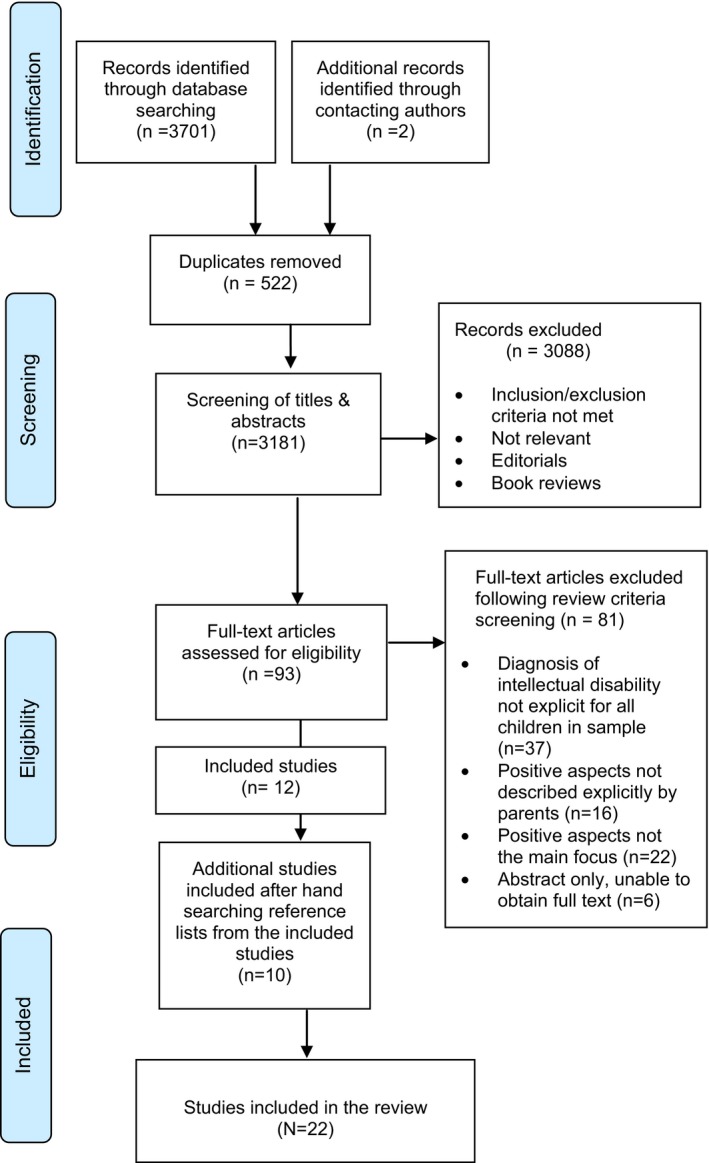
Flow diagram of the summary of the study selection process (Moher et al., 2015)

The initial database literature searches and contacting authors identified 3,703 articles for review. Following screening of the titles and abstracts and removal of duplicates, 93 full‐text articles were obtained and reference lists from these studies were also hand‐searched for other relevant studies (Greenhalgh, 2005) and eight additional articles were found. Twenty‐two studies were found to be eligible for inclusion and were retained following application of the review criteria screening criteria. The main reason why studies were excluded (*n* = 37) was due to the children in the sample not having a clear diagnosis of intellectual disabilities or the results not reported separately when included in a mixed sample of children with developmental disabilities. Supplementary file 1 shows the 81 studies which were excluded. Figure 1 shows a flow diagram of the study selection process.

### 4.1 Characteristics of the included studies

Characteristics of the included studies are shown in Table 4. These will be referred to by their assigned study number here forward and are categorized by study design.

**Table 4:** Characteristics of the included studies

	Authors/Country of origin	Aim of study	Instruments/methodology/questions to ascertain positive aspect/Term used to report the positives	(*N*)	Parental demographics	Child characteristics	Main positive aspects reported				Quality Assessment risk of bias
*Quantitative studies which reported only quantitative findings*											
1	Valentine et al. (1998) USA	The differences in African American and Caucasian mothers' experiences with their adult children with mental retardation and with service agencies	Modified burden and gratification scale for people with schizophrenia (Bulger et al., 1993) Measured both parental burden and gratifications Reports “gratifications”	71	Mothers only 13 Married (87%) Caucasian—60.6%(43), African American—39.4% (28)	Aged 22–55 years (mean age 33 years) Intellectual disability: Mild 32.4% Moderate 25.4% Severe 25.4% Profound 16.9%	Comparison of caregiver gratifications by race (T test scores)				Medium risk of bias
							Gratifications:	Caucasian	African American	*p* =	
							Appreciation for your work	2.84	2.77	0.49	
							Gives you pleasure	2.91	2.79	0.23	
							Feel good about yourself	2.68	2.80	0.46	
							Do a good job	2.45	2.81	0.02	
							Feel closer	2.77	2.88	0.32	
							Enjoy being with	2.87	2.96	0.19	
							Want to care more than a sense of duty	2.74	3.00	0.19	
2	Hastings et al (2002) United Kingdom	The associations between disability‐related maternal positive perceptions and the factors identified in previous family research as related to negative (i.e., stress) and positive (i.e., parental efficacy feelings)	Positive Contribution Scale (PCS), a subsection of the Kansas Inventory of Positive Perceptions (KIPP) (Behr et al., 1992) Scale measures the belief that the child with the disability has had a positive impact on the parent (1 = strongly disagree, 4 = strongly agree) **Reports “positive perceptions”**	41	Mothers only. Aged 30–59 years, Average age 41.40 years (*SD* 6.54) 36 (87%) married or cohabiting 19(46%) in paid employment 7% bachelor's degree, 46% no formal qualifications	*N* = 41 28 males, 12 females (one non‐response) 4–19 years, average age 11.9 years (SD3.88) “Children with learning disabilities”	The positive impact of the child itself (happiness and fulfilment) and its effect on the family in general (strength and family closeness) was positively predicted by the use of reframing coping strategies (*p* 0.001). Mothers reporting higher levels of caregiving demand reported more personal growth and maturity (*p* = 0.018) Reframing was also a positive predictor of positive effects on the mother herself (personal growth and maturity) (*p* = 0.035)				Medium risk of bias
3	Hastings, Beck, and Hill (2005) United Kingdom	1. Test the psychometric properties of the Positive Contributions Scale (PCS) to positive affect scale (PAS) 2. To compare perceptions of positive contributions reported by mothers and fathers	Positive Contribution Scale (PCS), a subsection of the Kansas Inventory of Positive Perceptions (KIPP) (Behr et al., 1992) Scale measures the belief that the child with the disability has had a positive impact on the parent (1 = strongly disagree, 4 = strongly agree) **Reports “positive contributions”**	200	142 families, 58 couples Mothers‐ 140 (70%) Mean age 39.42 years (*SD*7.33) 21% University education Father‐ 60 (30%) Mean age 42.08 years (*SD*6.98) 30% University education	*N* = 142 93 males 49 females 10.49 years average (*SD*4.01) Children with ID 22% (7) mild 59% (19) Moderate 13% (4) severe Rest undiagnosed 50% had additional physical disabilities	Mothers reported more positive perceptions than fathers on three of the scales: Learning through experience with special problems in life (*p* = 0.03) Source of strength and family closeness (*p* = 0.004) Expanded social network (*p* = 0.003) Fathers reported more positive perceptions than mothers on one scale: Source of pride and cooperation (*p* = 0.046)				Low risk of bias
4	Greer et al. (2006) Ireland	Four aims of which one relates to positive perceptions: Investigate whether behavioural and cognitive coping strategies predict levels of positive perceptions	Positive Contribution Scale (PCS), a subsection of the Kansas Inventory of Positive Perceptions (KIPP) (Behr et al., 1992) Scale measures the belief that the child with the disability has had a positive impact on the parent (1 = strongly disagree, 4 = strongly agree) **Reports “positive perceptions”**	36	Mothers only Aged 23–52 years (mean 37) 32 (89%) married 4 (11%) lone parents 58% homemakers, 11% unemployed, 34% in white collar jobs (47% of partners in white collar jobs) 11% no formal qualifications, 14% 3rd level qualifications	*N* = 36 *n* = 20 (56%) male, *n* = 16 (44%) female 5–8 years old (mean age 6 years) Intellectual disability: 31% Mild 53% Moderate 16% severe/profound	Agreement or strong agreement using self‐rated PCS scales on “the child is a…”: Source of happiness or fulfilment (78%) Source of strength and family closeness (75%) Source of personal growth and maturity (58%)				Medium risk of bias
5	Lakhani et al. (2013) Pakistan	The impact of caring for a child with mental retardation	Positive Contribution Scale (PCS), a subsection of the Kansas Inventory of Positive Perceptions (KIPP) (Behr et al., 1992) Scale measures the belief that the child with the disability has had a positive impact on the parent (1 = strongly disagree, 4 = strongly agree) **Reports “positive impact”**	54	Mothers only Age: 20–30 years (9%), 31–45 (63%) 46 years + (28%) 29% had a higher education degree 87% housewives	53% male 46% female Aged 6 and above. Mean age was 11.2 ± 2.62 years 54% ‐ mild to moderate mental retardation 46% severe retardation	Positive contribution subscales:	Mean	*SD*		Low risk of bias
							Learning through experience with special problems in life	3.39	0.19		
							Happiness and fulfilment	3.23	0.21		
							Strength and family closeness	3.17	0.26		
							Understanding life's purpose	3.22	0.75		
							Awareness of future issues	3.09	0.10		
							Personal growth and maturity	3.00	0.30		
							Expanded social network	2.43	0.55		
							Career or job growth	2.56	0.22		
							Pride and cooperation	2.44	0.23		
6	Vilaseca et al. (2013) Spain	Positive perceptions, anxiety and depression among mothers and fathers of children with intellectual disabilities	Positive Contribution Scale (PCS), a subsection of the Kansas Inventory of Positive Perceptions (KIPP) (Behr et al., 1992) Scale measures the belief that the child with the disability has had a positive impact on the parent (1 = strongly disagree, 4 = strongly agree) **Reports “positive perceptions”**	60	Mother/father married couples *Mothers:* Age 30 58 years (mean age 43.7 years) 93% Spanish origin 18.3% completed high school 23.3% F/T employment, 30% P/T employment *Fathers* Age: 35–59 years (mean age 45.3) 91.7% Spanish origin 50% completed high school 81% in full‐time employment	*N* = 60 35 male 25 female 1–6 years (10%) 6–12 years 20% 12–19 years (70%) Intellectual disabilities: 31.7% mild ID 35% moderate ID 33.3% severe	Mothers reported more positive perceptions than fathers on three PCS subscales: Strength and family closeness (*p* < 0.04) Personal growth and maturity (*p* < 0.02) Career/job growth (*p* < 0.05)				Low risk of bias
*Quantitative studies which reported both quantitative and findings from open‐ended questions/previous qualitative work*											
7	Scorgie et al (2001) Canada	Effective life management for families of children with Down's syndrome	Life management survey instrument (Scorgie et al., 1997) Parents ranked personal agreement/disagreement (1 = strongly disagree, 5 = strongly agree) for a range of transformational outcomes **Reports “transformations”**	53	39 mothers, 11 fathers, 2 foster parents, 1 guardian 84.9% dual parent homes	32 males 19 females Ages: 0–5 (9.8%) 6–12 (43.1%) 13–21 (25.5%) 21+ (21.6%) Mean 15 years All had Down's syndrome	**Strategies identified as important or essential to effective life management:** **Reframing:** Child brings traits such as joy, care, love of life and sensitivity towards others to their families and larger communities Developing skills to advocate on behalf of their child **Effective parent characteristics:** **Personal traits:** Maintaining a positive outlook, patience, willingness to grow and learn, determination (refusing to give up or persisting until an acceptable outcome is achieved) **Personal beliefs/philosophy of life:** “Life is what you make it,” having strong personal convictions, reliance on strong inner faith/religious convictions, belief in one's own inner strength **Transformational outcomes:** **Personal transformations:** Learned to speak out for their children, more compassionate towards others especially those in need, self‐empowerment, strength **Relational transformations:** Learned to see life from a different perspective, made a difference in the life of another person through advocacy **Perspectival transformations:** Learned what is really important and valuable in life and to cherish life, accept others non‐judgementally, help others				Medium risk of bias
8	Foster et al. (2010) USA	Demographic and psychosocial factors associated with well‐being including benefit finding	Modified benefit finding scale (Mohr et al., 1999) Open‐ended question: Do you have any other thoughts about your experiences caring for your child diagnosed with Smith‐Magenis syndrome that you wish to share at this time? **Reports “benefit finding”**	112	Mothers (*n* = 97) 87% Mean age 41.36 years (*SD* 9.60 years), 93.8% Caucasian 3% Latino/Hispanic, 2% African American/Black, 1% Multiracial, 77.3% married 92.7% had attended college Fathers (n = 15) 13% Mean age: 42.07 years (*SD* 9.85 years), 100% Caucasian, 100% married 84.6% had attended college	Average age reported: Mothers: 12.91 years (*SD* = 9.04). Fathers: 11.73 years (*SD* = 7.08) Gender of child: Mothers: Female 56 (58%) Male 40 (41%) Fathers Female: 8 (53%) Male 7 (47%) Child attending special school? Yes 75% mothers, 93% fathers	**Closer family relationships** Mothers 51 (53%) fathers 9 (60%) **Appreciating the preciousness of the child** Mothers 83 (87%) fathers 12 (80%) **Having a better perspective on life** Mothers 81 (84%) fathers 12 (80%) **Spiritual growth** Mothers 63 (65%) fathers 4 (27%) **Learning something about yourself (personal/emotional growth)** Mothers 74 (76%) fathers 10 (67%) Of 112 participants, 73 (65%) wrote in the open‐ended question section, the most common theme discussed was benefit finding/acceptance/gratitude/personal growth (41%).				Medium risk of bias
9	Skotko et al. (2011) USA	Family attitudes towards persons with Down's syndrome	Developed and piloted own survey instrument Parents asked to rate their level of agreement with statements about parental feelings towards their child on a Likert scale (1 = strongly disagree, 7 = strongly agree). Open‐ended question asking parents to share life lessons learned from their son or daughter who has Down syndrome Reports “positive parent outlook”	1989	63% mothers, 37% fathers – 88% married Average age 46.4 years (SD11.0) White 89%, Black/African American 2% Asian 2%, Other 7% “majority received a college/university degree or higher”	*N* = 1973 1,085 Males 888 Females <5 years – 33% 5–10 years 22% 10–15 years 15% 15–20 years 10% 20−25 years 8% 25−30 years 5% >30 years 7% All have Down's syndrome	Positive themes identified (*N* = 943):				Low risk of bias
							Personal self‐growth	48%			
							Patience	35%			
							Acceptance/Respect	24%			
							Love	24%			
							Joy	13%			
							Everyone has gifts/we're all more alike than different	12%			
							Lessons on blessings/faith/God	11%			
							Don't take anything for granted	8%			
							Kindness/empathy	8%			
							Perseverance	7%			
							Learning to advocate	6%			
							Learning how to be positive	5%			
							Tolerance	5%			
*Quantitative studies which reported only findings on positive aspects from open‐ended questions:*											
10	Kenny and McGilloway (2007) Australia	Assessing levels of caregiver strain, describing the practical day to day aspects of caring and the extent and nature of informal and formal support, exploring coping strategies employed by carers	Carer's questionnaire (McGilloway et al., 1995) Two open‐ended subsections for parents to describe the positive and negative aspects of caring **Reports “positive aspects”**	32	24 (75%) females All married Aged 28–57 (mean age 44 years) ½ employed outside the home, most of remainder ceased employment to care for their child	*N* = 32 19 (59%) male 13(41%)female Aged 2–17 (mean age 11 years *SD* 3.94): Mild 7/32 Moderate 19/32 Severe 4/32 44% had additional physical disabilities	child had brought positive changes into the carers' lives which, in turn had helped them to cope better less judgemental less materialistic and selfish than before their child was born more open and honest more confident more appreciative of the “little things in life.” more optimistic laughed more				Medium risk of bias
11	Rapanaro, Bartu, and Lee (2008) Australia	Perceived negative and positive outcomes reported by parents in relation to particularly stressful events and chronic caregiving demands encountered in the period of their son/daughter's transition into adulthood	Not provided Open‐ended question: Parents were asked to describe the negative and positive outcomes associated with the chronic demands of caring for their son or daughter in the past 12 months **Reports “benefits”**	119	107 (90%) females Mean age 48.05 years 77% lived in a metropolitan area 34% university degree, 33% school certificate, 12% “other” training, 20% no formal qualifications	70 male 49 female Aged 16–21 years Intellectual disabilities: 58.8% Mild 33.6% Moderate 7.6% Severe/profound	(a) Of the 94 parents who reported experiencing a particularly stressful event 45.7% (*n* = 43) reported a positive outcome or benefit. Three categories were identified: Enhanced personal resources/personal growth of parent (38.6%) Improvement in social support/relationships (38.6%) Enhanced personal resources/personal growth of son or daughter (22.8%) (b) In relation to the chronic demands of caregiving 64.7% (*n* = 77) reported perceived benefits or positive outcomes, four categories were identified: Sense of fulfilment and pride (52.9%) Personal growth/enhanced personal resources (35.6%) Enhanced social network (6.9%) Absence of certain care demands (4.6%)				
*Pluralistic evaluation:*											
12	Grant et al. (1998) United Kingdom	Preliminary findings of two instruments new to the field of Intellectual disabilities used for measuring caregiving rewards and stress.	Carers Assessment of Satisfaction Index (CASI) (Nolan et al., 1996) Measured factors which are perceived as a source of satisfaction and how much satisfaction is equated with each plus semi‐structured interviews. Questions not provided. **Reports “rewards”**	120	71% mothers, 9% fathers, 14% both parents, 6% other relatives ½ aged < 45 years, 45–64 (34%), >65 years (15%) 73% cohabitating, 27% single parents	½ children < 19, ½ >20 years 76 (63%) male 44 (37%) Female 51% unable to utter words or a few words only. 79% were able to make their needs known at least to family members	*Rewards emerging from the interpersonal dynamic (carer and the child)* Pleasure seeing relative happy, maintaining dignity of relative, expression of love, brought closer to relative, closer family ties, appreciation from others, relative does not complain *Rewards derived primarily from the intrapersonal orientation of the carer:* seeing needs attended to, seeing relative well turned out, knowing I've done my best, altruism, provides a challenge, feel needed/wanted, test own abilities, fulfilling duty, providing a purpose in life, stop feeling guilty *Rewards stemming from a desire to promote a positive outcome for the person with ID:* Help relative overcome difficulties, see small improvements in condition, keep relative out of institution, give best care possible, help reach full potential, developed new skills/abilities, less selfish, widened interests.				Medium risk of bias
*Qualitative studies:*											
13	Stainton and Besser (1998) Canada	Positive impacts [of caregiving]	Semi‐structured group interviews and constant comparative methods of analysis (Glaser & Strauss, 1967). “What are the positive impacts you feel your son or daughter with an intellectual disability has had on your family?” **Reports “positive impact”**	15	6 (40%) fathers 9 (60%) mothers Aged < 25–70. Mean age 50 years	Aged 0 to 35 years 7 (63%) female 4 (27%) male Self‐ reported degrees of intellectual disability ranged from low to high	Source of joy and happiness Increased sense of purpose and priorities Expanded personal and social networks and community involvement Increased spirituality Source of family unity and closeness Increased tolerance and understanding Personal growth and strength Positive impacts on others/community				Of Quality
14	Kearney and Griffin (2001) Australia	The experiences of parents who have children with significant developmental disability	A qualitative interpretative research approach underpinned by hermeneutic phenomenology (Van Manen, 1990) “Can you tell me your experience of living with (name of disabled child)” **Reports “joys”**	6	2 mother/father pairs and 2 mothers Two couples, 1 divorced, 1 mother separated	Age range 3–6 years 3 girls, 1 boy All children had major cognitive impairments	Child as a source of joy, love optimism Parents have become better people Have become stronger in the face of adversity New perspectives following overwhelming changes in personal beliefs and values				Of Quality
15	Scallan et al. (2011) Ireland	Exploration of the impact that a person with Williams syndrome can have on the family.	Semi‐structured interviews and a thematic analysis (Flick, 1998) Question not specified other than including the positive aspects of raising a child with Williams syndrome **Reports “positive impact”**	21	6 mother and father pairs, remainder mothers	*N* = 21 13 males 8 females Aged 4–43 years Mean age 20.9 years (*SD* 10.1 years) All have Williams syndrome	Positive Impact on siblings Joy brought by the person with WS Changed outlook on life Personal development Rewarding experience Friendships with other parents Company for parents Brings family closer				Of Quality
16	King et al. (2011)	To examine the nature of the benefits seen by parents of children with ASD and Down syndrome (differences between children at elementary and high school)*	Two semi‐structured interviews 2–5 months apart using a grounded theory approach (Strauss & Corbin, 1998) “Have your family values, priorities and worldviews changed over time?” “What sort of things do you celebrate?” **Reports “benefits”**	14	6 mother and father pairs 2 single mothers Aged mid−30s to mid−50s 6 couple's major urban homeowners. 2 major urban renters 3 finished high school, 9 college, 2 university	*N* = 8 3 boys, 1 girl ‐ Elementary school (ages 6–8 years) 3 boys, 1 girl ‐ High school (15–17 years) All have Down syndrome	*Parental level* Appreciation of the child for who they are Celebration of what the child can do *Family level* Appreciation of the family itself Appreciation of new opportunities Appreciation of learning for siblings and family *Societal level* Learning about differences, diversity and community Recognition of the capabilities of people with disabilities Benefits for other families of children with disabilities				Of quality
17	Kimura and Yamazaki (2013) Japan	Exploration of the lived experience of Japanese mothers who have delivered multiple children with intellectual disabilities	Semi‐structured interviews and interpretative phenomenological analysis (Smith, Flowers, & Larkin, 2009). “Please tell me your experiences of taking care of multiple children with intellectual disabilities” **Reports “positive experiences”**	10	Mothers only, All married Aged 35–50 years (mean age 41.7 years) 80% homemakers, 20% worked part time	*N* = 20 6 males, 4 females Aged 3 – 18 years (mean age 11.5 years) 8 males, 2 females Aged 0–13 years (mean age 7.4 years) All have intellectual disability	Parents were found to alter their perceptions about life by searching for positive aspects of caring. These came from three sources: *Themselves*: Provided them with confidence and optimism to overcome difficult situations, confronting each challenge, life has a meaning, *Others:* The importance of social/family support *The children with intellectual disabilities*: Finding positive features in their children and recognizing them as “treasures.” This metaphor was expressed with feelings such as grateful, cute, pleasure and participants looked back on their own lives and felt thankful and happy.				Of Quality
18	Thompson et al. (2014) United Kingdom	The caregiving impact of those who support a family member with intellectual disability and epilepsy.	Anonymous qualitative online survey comprised of twelve open‐ended questions exploring respondents' views on the needs of individuals with intellectual disability and epilepsy. Thematic analyses (Braun & Clarke, 2006) “In your experience how does having epilepsy and intellectual disability affect family life? What are the problems? What helps?” **Reports “positive experiences”**	42	No demographic details collected	No demographic details collected	The “positive impact” was identified as one of four thematic groupings: *Close families:* Close/loving family Family respond kindly to child “Few problems” [occasionally respondents indicated that they had few problems with care] Acceptance of/adaptation to limitations by siblings Personality shining *Supporting others:* Meeting/helping other families Siblings more patient/considerate people Involvement in activism Developed empathy for others *Changed perspectives:* Realized what is important in life Less paranoid about development of normal siblings Grateful for what they have				Lower quality
19	Beighton and Wills (2016) United Kingdom	Exploration what parents perceive to be the positive aspects of parenting their child with intellectual disabilities	Semi‐structured interviews and a thematic analysis (Braun & Clarke, 2006) “Can you describe to me in which ways [child's name] has had a positive impact on you or your family?” Reports “positive aspects”	19	14 mothers, 5 fathers Age range 29 – 68 years 58% Caucasian 58% married (*n* = 11) 37% retired (*n* = 7), 32% full‐time carers (*n* = 6) 19% Employed (*n* = 3), <1% Full‐time student (*n* = 1), 11% Unemployed (*n* = 2)	*N* = 19 42% Female (*n* = 8) 58% Male (=11) Age range 7–43 years Mild to severe intellectual disabilities	Seven key themes identified across all parents irrespective of gender or age of the child: Increased personal strength Changed priorities Greater appreciation of life The child's accomplishments Increased spirituality/Faith More meaningful relationships with others The positive effect the child has on others				Of Quality
*Mixed methodology*											
20	Adithyan et al (2017) Southern India	Impacts on the caregivers of children with intellectual disability	National Institute for the Mentally Handicapped ‐ Disability Impact Scale (Peshawaria, 2000). Scale was administered to study the negative impacts only. Positive impacts were ascertained from focus groups and in‐depth interviews. Type of thematic analysis undertaken not provided “What were the good changes that have happened to you since this child came into your life?” **Reports “positive impacts”**	22	21 Mothers, 1 Father Mean age of parents 40.5 years	*N* = 22 68% Males “Most children aged > 10 years” 62% of children were diagnosed with “multiple disabilities,” most often cerebral palsy (30%) along with intellectual disability	Three main areas identified: Increased self‐esteem Strengthening of family ties Increased social responsibility				Low risk of bias
*Case study*											
21	Durà‐Vilà et al. (2010) United Kingdom	Explore how the unexpected experience of an unusual offspring is attributed to sacred religious meaning	Semi‐structured face‐to‐face interviews undertaken to produce two illustrative case reports **Reports “gains”**	2	Two mothers, one father	One boy with Down's syndrome aged 16 One girl with severe intellectual disabilities aged 9	Child brings meaning to life God sent child because mother needed him Blessing from God Strengthened marriage We love her so much Brings us good things, a good luck charm				Of quality
*Retrospective review*											
22	Wikler et al. (1983) USA	The author is reporting a previously discounted “positive” finding from a study they had undertaken which explored adjustment in families with a mentally retarded child (Wikler et al., 1981)	Original study ‐ Questionnaire survey **Reports “strengths”**	32	No details provided	No details provided	75% (*n* = 27) of parents reported they had become “stronger,” of these 46% (*n* = 12) felt “much stronger”				(Original study ‐ medium risk of bias)

Seven qualitative studies were included, one was pluralistic evaluation which illustrated the quantitative findings with a qualitative case study and one used a mixed methodology study which ascertained the positive aspects from only qualitative interviews. Although eleven quantitative studies were included, four included open‐ended questions with two reporting both the quantitative and qualitative responses (8,9) and two reported just the findings from the open‐ended questions (10,11). One quantitative study supplemented their results with quotes taken from an earlier qualitative study (7), and a review was included in which the authors reported a previously discounted “positive” finding from a study they had undertaken which explored chronic sorrow in families with a mentally retarded child (22).

The majority of studies originated from high‐income countries, with two undertaken in low‐and middle‐income countries, Pakistan and Southern India (5, 19). One UK study obtained responses from seven countries although most (41/48) were predominantly from within the United Kingdom (18).

The most common recruitment method was through state/county service agencies (1, 4, 11,16,17,18,21). Some studies drew samples from larger studies which explored care packages, good life management and family attitudes towards a person with Down's syndrome (7, 9, 12). Others recruited from special schools (2,3,6) and support groups (8, 10,13, 15,19), approached parents that they had worked with previously via mail out (14) and recruited from a private day care centre (5).

Sample sizes of the quantitative studies ranged from 32 to 1,989 parents (10 and 9 respectively); however, the former study only reported findings from the open‐ended questions. All except two of the remaining quantitative studies (2,4) contained a sample size of 50 or more participants. Response rates were provided in all but one study (10) with the remaining studies reporting overall response rates of between 5% (71/1352) and 78% (156/200) (1,3, respectively). Although having a low response rate, the former study attempted to recruit parents by contacting every parent of an adult child on the register of two county disability service agencies. Two studies acknowledged their methods of recruitment or low response rates as a limitation (2,8).

### 4.2 Methodologies and methods of the included studies

Table 4 shows the methodologies, instruments/scales and questions used to measure and ascertain the positive aspects. It also shows the many varied terms the authors used to report the positive aspects. All eleven quantitative studies, the pluralistic evaluation and mixed methodology study were of a cross‐sectional design using questionnaire surveys which were self‐administered except for three which used a researcher to complete the questionnaire with the participant (1,4,5). The most commonly used scale was the Positive Contribution Scale (PCS), a subsection of the Kansas Inventory of Positive Perceptions (KIPP) designed specifically to assess the families' cognitive perceptions about their child with a disability (Behr, Murphy, & Summers, 1992). The scale consists of nine independent subsections, and although administered in five studies (2,3,4,5,6), three studies measured only three of the nine subsections (2,4,6).

Six of these studies had at least one primary aim that focussed on either exploring the parent's positive perceptions of the positive contribution the child had brought to the parent or family or the factors relating to positive outcomes, that is coping differences in perceived positives between mothers and fathers (2, 3, 4, 6, 8,11). One study explored the phenomenon in mothers who had multiple children with intellectual disabilities (17).

Three qualitative studies had primary aims to examine or explore the positive aspects of caring and used positively phrased questions (13,19,20) whilst four explored the experiences or impact by asking similar neutral questions (14, 16,17,18,21). The questions were not specified in two studies (12, 15).

### 4.3 Quality of the included studies

The quality grading of the studies is shown in Table 4 as an overall assessment. In the quantitative studies, validity and reliability were affected in studies considered to be of a medium risk of bias as they did not report methods clearly enough to replicate the study (12), used modified instruments without explanations of what was changed or details of piloting (8,10). The two studies that had small sample sizes might lack statistical power, and therefore, their results should be viewed with caution (2,4). Studies also included selection/sample bias, that is recruiting friends and acquaintances of the first author (10) and staff selecting “good copers” to participate (7). Questionnaire surveys completed by researchers for parents may have also introduced social desirability bias (1,4,5), and an honorarium was paid for return of the completed questionnaires distributed at a specialist school (3).

Six qualitative studies and the mixed methodology study (20) were considered to be of quality as all had some degree of reflexivity about why they were investigating this topic and had clear research questions, aims and theoretical underpinnings for the qualitative methodologies used. Reliability was demonstrated by detailed Methods sections, and three studies described member‐checking of the analyses by other researchers (13,15,19,21) with the latter two studies sending out the transcripts for checking by the parents for accuracy and comments. Three did not, however, fully describe the participants' characteristics (13,14,15), and another introduced selection bias as they relied on parent group leaders and service providers to nominate families who would fit the criteria and be able provide a wide range of perspectives (16). The pluralistic evaluation (12) was considered to be of lower quality as the quantitative findings were supplemented by only one qualitative case study, a limitation acknowledged by the authors.

### 4.4 Theoretical frameworks

The majority of studies discussed findings of positive perceptions in relation to stress, burden, coping and adaptation although not explicitly presenting a theoretical framework. All except two of the studies were atheoretical (4,5). Study 4 used an adapted version of the “working model for the study of families positive perceptions” (Hastings & Taunt, 2002). However, their findings showed that this model had reduced explanatory powers and proposed that the findings instead provided evidence for “positive psychology.” Study 5 proposed the two‐factor model of caregiving to explain the coexistence of positive and negative outcomes (Lawton, Moss, Kleban, Glicksman, & Rovine, 1991).

## 5 SYNTHESIS OF THE RESULTS

Because parent or child characteristics may influence whether and the extent to which positive aspects of caring may be reported, these are summarized here:

### 5.1 Parental and child characteristics

The parents were predominantly married/cohabiting Caucasian mothers, the majority being between 35 and 50 years of age. Five studies contained mothers only (1,2,4,5,17), and three studies contained a small number of non‐biological parents, but results were not broken down to explore differences in parental positivity (4,7,9). None of the studies reported the length of time the parents (biological, adoptive parents, guardians and stepmothers) had supported the child but it cannot be assumed they parented them from birth. Although income details were provided in four studies (5,8,9,13), these were not comparable due to the age of the studies and being from different countries. As a high proportion of parents attended higher education institutions and were employed outside the home, it may be assumed that they were from higher socio‐economic groups.

The ages of the children they parented ranged from birth to age 55. The majority of studies were conducted with parents of children aged 19 years and under (mean/average age ranged from 3 to 11 years) (2,3,4,5,6,8,10,11,14,16,17,21), six contained both children and adult children (7,9,12,13,15,19), and one contained adult children only (1). No child characteristics were provided in three studies (18,20,22), and the latter study also failed to provide any details of the parents. Most studies contained a higher ratio of male children than female children with the majority having moderate to severe intellectual disabilities.

### 5.2 Positive aspects

As shown in Table 4, twelve different terms were used by the authors to report the positive aspects, the most common being positive impact(s) (5,13,15,18,20). Others refer to positive perceptions (2, 4,6), benefits or benefit finding (8,11), positive aspect(s) (10, 14,18), positive contributions (3), rewards/satisfactions (12), positive experiences (16), gratifications (1), gains (21), strength (22) and transformations (7). Another refers more generally to the parents' outlook on life being more positive because of their child (9). Study 16 used both the terms positive contributions and benefits interchangeably throughout. Of the seven quantitative studies which collected data from scales/instruments, only two reported the positive aspects as they were described in the measurement scale (1,3). Although five studies used the PCS scale, only one reported the findings as positive contributions, whilst three reported positive perceptions (2,4,6) and the other as positive impacts (5).

Only two studies were consistent in their operational definition and reported the positive aspect similarly. Study 11 defined what they were exploring as “the belief or conclusion that an adverse event or circumstance has revealed or evoked positive outcomes in one's life” (McCausland & Pakenham, 2003:854) and reported benefit finding. Study 1 (p580) used the “overall pleasure, satisfaction and sense of mental well‐being that a person receives in her role as a caregiving parent” and reported gratifications.

### 5.3 Thematic analysis

A narrative synthesis which synthesizes common elements across otherwise heterogeneous studies was undertaken. This method involves using a textual narrative approach and thematic analysis, which is particularly suitable for identifying the main, recurrent and or most important themes (based on the review question) and provides a possible structure for new research (Lucas et al., 2007). The characteristics of positive aspects reported in both qualitative and quantitative primary research studies were summarized using words and text within individual studies and then between all included studies. These data were then grouped into eight themes (Thomas et al., 2012), and the results are shown in Table 5.

**Table 5:** Positive aspects identified across the studies

Theme	Positive aspects identified	Studies reporting		
		Quantitative	Qualitative	Pluralistic Evaluation/mixed methods/case study/Review
Personal strength	Confidence/more confident, personal growth, strength, personal growth and maturity, increased sense of purpose, more perseverance, more determined (refusing to give up until an acceptable outcome is achieved), belief in one's own inner strength, confronting each challenge.	2,3,4,5,6,7,89,11	13,14,15,18,19	22
Personal development	Becoming a better person, being less materialistic and selfish, more tolerance and patience, more open and honest, increased self‐esteem, kinder, learning something about yourself, feeling good about yourself, learning to be positive, sense of fulfilment and pride, more hopeful, more optimistic, laugh more, career/job growth, developed new skills and made a difference through advocacy	1,3,5,6,7,8,910,11	13, 14,15, 16,17,18,19	12,20
New outlook or perspective on life	Changed focus of personal priorities about what is important in life, gives meaning to life, learned how to see life from a different perspective, appreciative of the little/small things in life, widened interests, grateful for what they have, increased social responsibility, cherish life, overwhelming changes in personal beliefs and values, less judgemental, and selfish, increased sensitivity, empathy, tolerance, kindness, compassion and understanding towards others in need or for those who have disabilities, more patience, acceptance of others without judgement	1,3,5,6,7,8,9,11,14	13,14,15,16,17,18,19	20,21
The child as a source of happiness and fulfilment	Child is a source of joy, happiness, pleasure, pride and fulfilment, enjoying being with the child, have a deep personal bond unlike any other, appreciating the “preciousness” of the child, grateful and lucky to have their children, the child is a source/expression of love and happiness, company for the parents, personal growth or improvements in their child's condition, unexpected achievements/accomplishments, small improvements in condition, watching the child achieve things never thought possible, child is a good luck charm	1,2,3,4,5,7, 9,10,11	13,14,15,16,17,18,19	12,20,21
Improved/expanded relationships	Increased family unity and closeness, support and expanded community networks/involvement, closer family ties/relationships, more meaningful relationships with others, improved social support/friendships, interacted and socialized with others they would otherwise have not met (i.e., parents of children with intellectual disabilities) meeting/helping other families, stronger marriage	2,3,4,5,6,8,11	13,15,16,17,18	12,20,21
The positive effect the child has on others	Child has a positive impact on siblings, siblings had become more caring, accepting, sensitive, understanding, considerate and mature, the child has a positive effect on others in the community, the child bringing joy to others.	7,8,9,10,11	13,15,17,18,19	20
Increased spirituality/Religiosity	Stronger inner faith and religious convictions, increased spiritual growth, child is blessing/angel from God,	7,8,9	13,19	12,21
Caring role	Keeping the child out of an institution, seeing them well turned out, helping them overcome difficulties, helping them to reach their full potential, doing a good job, gaining appreciation for their work, appreciation from the child and other family members, wanting to care more than a sense of duty, altruism, parenting role being a rewarding experience, pleasure seeing the child happy, maintaining dignity of the relative, seeing their needs attended to, feeling needed/wanted, testing own abilities, fulfilling duty, providing a purpose in life, giving the best care possible, a rewarding experience, absence of care demands	1,11	15	12

## 6 FACTORS RELATING TO PARENTAL POSITIVITY

Only two studies purposely investigated factors which predict the likelihood of a parent experiencing positive aspects (2,4). From other studies, other factors were identified which were found to have a relationship to parental positivity:

### 6.1 Child characteristics

Certain child characteristics were found to affect the parent's ability to perceive things as positive. Parents whose children had fewer medical problems and learning difficulties reported a more positive outlook (11). A significant negative correlation was found between the severity of the child's behaviour, emotional problems, perceived care demand and the ability to identify positive aspects (4). A significant negative correlation was found in mothers with the greater the intellectual disability, the smaller number the positive perceptions reported, which was not evident in fathers (6). In contrast, the increased difficulty of care required for the child and mothers with higher caring demands emerged as a significant positive predictor for parents reporting personal growth and maturity in two studies (2,3). The studies contained children who had a range of intellectual disabilities; however, it should be recognized that they are not a homogenous group and it could be that different social and psychological factors are at play across different behavioural phenotypes. Previous research has reported that parents of children with Down's syndrome experience more rewards compared to parents of children with other disabilities (Hodnapp et al., 2001). However, in the studies that contained samples of children who had Down's syndrome (7,9,16) the positives identified were similar to those identified in the other studies with one small exception; in two studies, improved or expanded relationships with others were not identified as a positive, but this may be due to the wording of the questions asked (7,9). No differences were found in the study of parents of children with Down's syndrome and a comparison group of parents who have children with autism spectrum disorder (16).

### 6.2 Culture

Despite a reported national culture of stigma and blame about having a child with an intellectual disability in these countries, mothers in Pakistan, Southern India and Japan all reported that their children were a source of happiness and fulfilment, they had gained a new perspective on life, and they had gained increased or expanded social networks (5,17,18). For mothers in Pakistan, having a new outlook or changed perspective on life was found to be the highest scoring subscale on the PCS (5), and similarly, in southern India parents reported as a positive that they had now gained understanding of the sufferings of children with similar conditions (20).

African American mothers reported slightly higher levels of gratifications and positive impacts than Caucasian/Anglo mothers after controlling for socio‐economic status (educational levels and income) (1), and parent's who were of Hispanic origin and/or of a lower educational background who had children with Down's syndrome were more likely to report a positive outlook (9).

### 6.3 Gender of the parent

Although the majority of the studies contained mothers, statistically significant differences between mothers and fathers in terms of total PCS scores were found. In the PCS subscale most closely related to having a new outlook or changed perspective on life, there was a statistically significant difference as mothers reported more positive perceptions than fathers (*p* = 0.03) (3). No difference was found in the child being a source of happiness and fulfilment between mothers and fathers although fathers were more likely to perceive their child as a source of pride and cooperation than mothers (3,6), although this was not statistically significant in the latter study. From the quantitative studies with open‐ended questions (8,9), three‐quarters of mothers and two‐thirds of fathers reported experiencing personal/emotional growth and just under half of parents (435/997) reported increased personal self‐growth as a result of having their child with Down's syndrome.

### 6.4 Social support

The helpfulness of informal social support services and acquiring social support were found to be predictors of maternal positive perceptions (2), and having a husband or parent available to share the burden was found to have a significant influence on altering the mother's outlook to a positive one (17). All but four study parents reported improved and strengthened relationships with others, and this could lead to increased social support for both the parent and their child.

### 6.5 Mental well‐being

Higher levels of mental well‐being and self‐efficacy (*p* < 0.001) were associated with increased benefit finding in both mothers (*p* < 0.05) and fathers (*p* < 0.01) (8), and positive perceptions were found to coexist with symptoms of anxiety and depression in both fathers and mothers (6).

## 7 DISCUSSION

This is the first review of the positive aspects of parenting a child with intellectual disabilities as reported by parents/carers. Consistent themes were identified although the studies were conducted over a number of countries and used differing methodologies. Capturing these positive aspects was less straightforward as twelve different terms were used to describe the construct and only two studies were underpinned by a clear theoretical framework. This resulted in a wide variety of instruments and questions being used to measure and describe the construct. Although no clear definition was employed across studies, similar themes were consistently reported and these consistent themes as a whole comprise the construct of a positive aspect of parenting a child with intellectual disabilities. The positive aspects described were pervasive and diverse, and the majority of themes identified were related to intrapersonal factors derived from the orientation of the parent. These include personal growth/strength, personal development, having a changed outlook on life, having expanded relationships with others, their child being a source of happiness and fulfilment, having increased spirituality/religiosity and the nature of the caring role. These are consistent with those identified in other carer studies. The positive effect their child with intellectual disabilities has on others was identified as a positive aspect and unique to these parents.

All except one of the studies included the review are from the last two decades. This is most likely because in most early studies positive aspects were not the focus combined with a shift away from placing children in institutional care and also a rise in the positive psychology movement from the 1990s. This is illustrated by the early study included in this review whereby the positive findings were initially discounted in a study exploring chronic sorrow (Wikler, Wasow, & Hatfield, 1981), with the authors later explaining that this was an example of a pervasive stance adopted among professionals in which the problems instead of the strengths were the focus (22).

Positive aspects were identified from both open‐ended neutral questions in addition to those measured purposely. Whilst there were consistent positive aspects identified across all studies, those relating to increased religiosity/spirituality and the positive effect the child has on others were identified only in the qualitative studies, the quantitative studies with open‐ended questions and the study which specifically explored sacred meaning (21). This theme was not captured by quantitative measures. This theme formed the basis for a PCS subscale “source of understanding life purpose”; however, two studies chose not to include this subscale in their survey (2,4) and another (3) discounted the scale after data collection as the questions were “rarely seen as applicable” by parents, and therefore would have resulted in poor internal consistency (p162). This highlights that positive aspects are influenced by the ways in which the construct is measured yet the measurement tools themselves are informed by different epistemological views and models. This lack of clarity has led to a number of terms that are being used synonymously which appear to describe the same construct and some of which may be interrelated. It also indicates that objective measures alone are not sufficient to fully represent positive aspects in its entirety, consistent with the findings of Jess, Hastings, and Totsika (2017).

No differences were found between mothers and fathers reporting positive aspects. The parents included in the studies in this review were, however, predominantly married/cohabiting white mothers from a high socio‐economic status. Due to the limited number of studies focusing on couples, the data obtained from mothers only provide an incomplete picture of parenting a child with an intellectual disability as fathers can have a different role within the family and may have a different relationship with the child. The high number of married mothers as participants could also mean that they are likely to have more social support from their spouse during times of stress and report feeling more positive, consistent with the stress‐buffering hypothesis (Cohen & McKay, 1984). However, although a positive was that the child was perceived to have strengthened the parent's marriage, in contrast, 11% (104/943) of parents felt that their child with Down's syndrome had “uniquely” put a strain on their marriage (9). There could be differences in positive perceptions between lone parents and those who are married which would benefit from further exploration. Similarly, having higher socio‐economic status may allow these parents the opportunity to pay for additional support and services, which may allow them to perceive things in a more positive light.

All but two of the studies were conducted in high‐income countries where considerable support services are provided for families (Families Special Interest Research Group of IASSID, 2014). This is in contrast to low‐ or middle‐income countries where <50% provide any support to families of children with intellectual disabilities in areas such as respite care, in‐home support or advocacy (WHO, 2007).

Only studies published in English language were included, and the parents were predominantly Caucasian, which has limited the consideration of the contribution of different cultural traditions and expectations and which merits further investigation as ethnicity was found to have an influence on parental positive aspects. Five quantitative studies used the PCS subscale of “strength and family closeness” (2,3,4,5,6); however, although most were in agreement or strong agreement that their child was a source of strength and family closeness (4), the mean score for family closeness in mothers from Pakistan ranked very low, seventh out of nine subscales (5). The findings of study 1 were consistent with studies of Latina mothers of children with intellectual disabilities (that did not meet the inclusion criteria), which report slightly higher levels of gratifications and positive impacts than Anglo mothers (Blacher & Baker, 2007; Blacher, Begum, Marcoulides, & Baker, 2013).

A theme found in almost all studies was personal growth and personal development and could relate to parents developing/gaining increased self‐efficacy and/or self‐esteem. Parental self‐efficacy was found to have the strongest bearing on maternal positivity in mothers of children with intellectual disabilities (Jess et al., 2017). Having high self‐efficacy enables parents to exercise control over their lives, a prerequisite for problem‐focused coping, and plays a significant role in health behaviours as low self‐efficacy is associated with depression, anxiety and helplessness, whereas conversely having high self‐efficacy is related to psychological well‐being (Bandura, 1993).

Reporting improved and strengthened relationships with others could lead to increased social support for the parent. In parents of children with developmental disabilities, both social support and positive reframing coping strategies have been found to be influential mechanisms through which optimism influences “benefit finding” (Slattery, McMahon, & Gallagher, 2017). Social support is reported to be the strongest and most frequently used strategy in high coping families which contain a child with intellectual disabilities (Taanila, Syrjälä, Kokkonen, & Järvelin, 2002). This support may provide parents with the opportunity for discussion and to make meaning of their situation (Lepore & Evans, 1996), directly enhance mental well‐being and strengthen feelings of self‐esteem and self‐efficacy (Cohen & Wills, 1985). It is also suggested that interpersonal relationships may provide positive experiences that promote growth by bolstering positive affect (Cadell, Regehr, & Hemsworth, 2003). Religion and spiritual beliefs have been found to provide an interpretative framework to understand, accept and view a child's disability in a more positive light (Durà‐Vilà, Dein, & Hodes, 2010) and could also relate to increased social support gained from others, for example members of a church. In general studies, religiosity has been associated with benefit finding and strongly related to stress‐related growth (Helgeson, Reynolds, & Tomich, 2006; Pargament, Smith, Koenig, & Perez, 1998; Park, Cohen, & Murch, 1996; Skinner, Correa, Skinner, & Bailey, 2001).

None of the studies in this review contained a comparison group of typically developing children with two studies acknowledging this limitation (9,15). Studies that have explored positive impacts in relation to parental ethnicity and which also included a control group of typically developing children concluded that parents of children with intellectual disability report the same types of positive perceptions of childrearing experiences as parents of typically developing children (Blacher & Baker, 2007). The parents in studies in this review described positive emotions such as joy, happiness, pride and love (Folkman & Moskowitz, 2004; Fredrickson, 2011), and when their child achieved something unexpected, they perceived this to be positive. However, similar themes have also been described as the joy of parenting a typically developing child (Brooks, 2013), and therefore, it is not possible to confirm whether the child being a source of happiness and fulfilment is an exclusive phenomenon of parenting a child with intellectual disabilities or whether it could be construed as common to the majority of parents describing their child and whether it is influenced by the sociocultural context (Blacher & Baker, 2007). This merits further investigation.

No studies included in this review reported post‐traumatic growth (PTG) although one study explored benefit finding following adversity (8). Interestingly, Tedeschi and Calhoun (2013) who coined the term PTG initially used the terms “perceived benefits” and “positive aspects” but felt that PTG captured the essentials of the phenomenon better as it made it clear that the person had developed and grown beyond their previous level of adaptation, psychological functioning or life awareness. Researchers exploring positive aspects in families of children with intellectual disabilities may therefore have avoided using the term PTG to prevent the parent feeling stigmatized as trauma refers primarily to receiving damage or injury and highlights the negative effects of an event. Tedeschi and Calhoun (2013:54) themselves, also advise caution; “…because of the potential for misunderstanding it may not always be a good idea to use that term [posttraumatic growth] with clients.” However, in studies of survivors exploring PTG following various traumatic events, very similar themes are reported to the positives identified by parents in this review; a positive change in the persons sense of self, changes in relationships and spiritual growth have been reported (Joseph & Butler, 2010; Tedeschi & Calhoun, 2004). These studies also emphasize that the presence of growth does not imply the absence of pain and distress. Further exploration of growth following adversity using an associated theoretical model in this parent would provide clarification.

Similarly, no studies were included relating to positive reframing/positive reappraisal, despite this being a commonly used strategy used in benefit finding (Helgeson et al., 2006). However, these are coping strategies and the aim of the review was to explore what parents describe to be positive. However, it should be noted that statistically significant relationships have been found between positive reappraisal coping and PTG (Park et al., 1996; Urcuyo et al., 2005; Zoellner & Maercker, 2006). Similarly, meaning‐making coping whereby a person gains greater control and is better able to look at events or a problem in a different way is reported to promote positive growth (Cadell et al., 2014).

All of the studies included were limited by their cross‐sectional nature which provides a static view of parenting when caring is dynamic over the lifespan. Therefore, this review could provide only a limited understanding of any association between length of caring and reporting of positive aspects or benefit finding. The majority of studies included parents of children under the age of 19 years, and only one study contained a sample of only adult children (1). In this and another study in which half of the participants were adult children (12), positive aspects were identified; however, these mostly related to undertaking caring duties for their adult child to a high standard with the concomitant appreciation by the child and other family members. Another study which included children aged 4–43 years described how looking after their child was a rewarding experience (15) but this was in contrast to parents of younger adults aged 16–21 years who considered the “absence of certain care demands” as a positive (11). The former studies are twenty years old, and it could be speculated that these parents are reporting positives as they wanted to keep their children at home and not be placed in a long stay institution as some of these parents may have been advised to do when their children were younger.

The children in the review were aged from birth to age 55 years, but evidence was scant if there were any differences in the type of positive aspects identified by parents and the child's age. Study 19 found no difference in the type of positive aspects identified by the parents across the child's age and when asked parents provided mixed responses as to whether they felt these changed over time. It could be hypothesized that as the child ages, complex behaviours and medical conditions could worsen alongside the realization of the need for lifelong dependence which may affect the parent's ability to perceive positives. However, study 16 reported that families with older children (ages 15–17) were more likely to see positive aspects of their situation than those with younger children (6–8 years). In many studies relating to adversity, there is mixed evidence as to when any benefit finding occurs (Helgeson et al., 2006; Joseph & Linley, 2005; Picoraro, Womer, Kazak, & Feudtner, 2014; Zoellner & Maercker, 2006). It has also been argued that benefit finding is superficial and transient in nature (Tennen & Affleck, 2009) as opposed to PTG which results in changes to a person's core schema (Janoff‐Bulman, 2006). Further research is needed that explores if differences exist in the types of positives identified between parents of younger and older children and would help to establish if, and/or when, parent's positive perceptions change over the parenting/caring lifespan and at key transition stages such as retirement. Similarly, longitudinal studies could aid understanding if parents are experiencing initial benefit finding (considered to be a positive reframing strategy), then PTG over time, or if they develop a repertoire of adaptive coping strategies as the child ages as it may possible that different constructs are being described by these parents. However, the use of all three constructs has been shown to increase eudaimonic mental well‐being and therefore beneficial to these parents (Carver, Lechner, & Antoni, 2009; Joseph, Murphy, & Regel, 2012).

Only one study reported a significant negative correlation between the number of positive perceptions reported and the child having more severe intellectual disabilities (6), and therefore, this review was not able to confirm associations found in studies of adversity that greater levels of trauma (severity of intellectual disability) are associated with higher levels of benefit finding (Linley, 2004). Similarly, it was not possible to ascertain whether there was a curvilinear relationship with parents who report the most benefit finding experiencing a moderate rather than a low level of exposure to trauma (Helgeson et al., 2006).

The majority of studies were not initially theoretically grounded, but the findings of two studies (12,19) confirmed the validity of the transactional model of stress (TMS) (Lazarus & Folkman, 1987) concluding that many of the positive aspects/satisfactions described were linked to, or outcomes of, successful strategies of cognitive coping (meaning making). Positive reframing coping strategies were found to be a significant independent predictor for maternal positive perceptions by Hastings, Allen, McDermott, and Still (2002). This model was also considered to be a suitable theoretical framework to underpin the parents' positivity due to its emphasis on meaning‐focused coping which includes positive reappraisal/reframing coping strategies (19). Study 4 initially used an adapted version of the “working model for the study of families' positive perceptions” (Hastings & Taunt, 2002). However, their findings showed that this model had reduced explanatory powers and proposed that the findings instead provided evidence for “positive psychology” of which positive reframing and meaning making are two key aspects, along with positive relationships, positive emotions and pursuing mastery (Seligman & Csikszentmihalyi, 2000). This was consistent with study 14 who concluded that parents were constructing meaning (Frankl, 1963). Another suggested support for the social constructivist theory of disability (13) (Oliver, 1990). Both benefit finding and PTG are underpinned by positive psychology, and further research which explores growth from this theoretical lens would increase understanding.

This review has highlighted several knowledge gaps which could aid work to support parents. Positive aspects are not consistently defined although are able to be identified. Clarification of this as a construct would lead to improved and consistent theoretical frameworks, methodologies and measurements being used and allow for more comprehensive comparisons of the findings to be made both within and across other carer groups. Identifying predictors of positive aspects (including those identified in this review) would be beneficial. This could guide professionals in providing support and identify early interventions including reframing strategies which may enhance parental positivity and improve mental well‐being.

## 8 LIMITATIONS

This review has revealed considerable heterogeneity of study designs and definitions which necessitated a rigorous and transparent narrative synthesis. Nevertheless, there are limitations.

A number of different terms were found to be used to describe positive aspects, and this difficulty of searching for positive aspects was also acknowledged in a systematic review of positive experiences of caregiving in stroke (Mackenzie & Greenwood, 2012). In addition, due to the wide range of terms used to describe an intellectual disability, despite rigorous searching, some studies might have been missed. Similarly, some early publications may not have been found on searching as keywords were only uniformly adopted by journals after c. 2000.

There is far more evidence than is reported here that parents do have positive experiences. However, many studies were excluded due to the positive aspects being a dependent variable in quantitative papers. Another reason is that in some studies the children have disabilities or developmental disabilities (such as autism), but it was not stated that the children also have an intellectual disability therefore again were excluded.

Most of the studies recruited parents through service agencies or support groups who support people who have a learning disability. This is a common approach to research with this parent group as the low prevalence of intellectual disability in the population makes recruitment “notoriously difficult” and impractical to randomly sample from the population in order to obtain a sufficiently large sample (Gallagher, Phillips, Oliver, & Carroll, 2008:21). However, recruiting in this way can introduce selection bias because those parents who might experience stress would be more likely to attend support groups and some parents who do not attend might have differing perspectives. In addition, parents who may not be able to identify any positive aspects may not have volunteered to participate, limiting the generalisability of the findings.

## 9 CONCLUSION

There is an existing body of evidence about the positive aspects of caring, but this is the first systematic review to critically examine what parents describe to be positive about parenting their child who has intellectual disabilities. Positive aspects are not consistently defined although are able to be identified even in studies lacking clear operational definitions and consistent means of measurement. From both the quantitative and qualitative studies, similar themes relating to positive change, growth and development largely related to parental intrapersonal orientation and individual characteristics are reported.

Positive aspects were variously attributed to theories relating to coping, adaptation or growth following adversity but no single theoretical framework underpinning this parental positivity emerged from the review, although a small number of studies related their findings to positive psychology. Several studies emphasize that the presence of growth or positive change does not imply the absence of pain or distress.

This body of evidence highlights the importance of both professionals and parents being aware that there are positive aspects in addition to the stresses associated with parenting a child with intellectual disabilities.
