# Supplementary material for: How parents describe the positive aspects of parenting their child who has intellectual disabilities: A systematic review and narrative synthesis
Source: J Appl Res Intellect Disabil. 2019 May 20;32(5):1255–79. doi: 10.1111/jar.12617 (PMC6852490; doi:10.1111/jar.12617)
Supplement: Supplementary file 1 [file JAR-32-1255-s001.docx]

**STUDIES EXCLUDED AFTER FULL TEXT SCREENING (N=81)**

**ID not primary diagnosis/mixed sample of IDD (n=37)**

Behr & Murphy, (1993)

Bekhet et al. (2012)

Boström et al. (2011)

Burton-Smith, Burton‐Smith, & McVilly, (2009)

Cairns et al. (2014)

Davis, Nolen-Hoeksema, & Larson, (1998)

Dillenburger & McKerr, (2011)

Findler, (2014)

Green (2007)

Glidden & Natcher, (2009)

Gupta & Singhal (2004)

Hastings et al. (2005)

Hastings & Taunt, (2002)

Kayfitz et al. (2010)

King et al. (2012)

Konrad, (2006)

Krauss et al. (2005)

Manor-Binyamini, (2014)

Manor-Binyamini, (2016)

McConnell et al. (2015)

Mullins, (1987)

Neely-Barnes & Dia (2008)

Nelson, (2002)

Pelchat et al. (2003)

Phelps et al. (2009)

Picoraro et al. (2014)

Poehlmann, Clements, Abbeduto, & Farsad, (2005)

Sarriá & Pozo (2015)

Scorgie & Sobsey (2000)

Scorgie & Wilgosh (2008)

Slattery, McMahon, & Gallagher, (2017)

Strecker, Hazelwood, & Shakespeare-Finch, (2014)

Trute et al. (2007)

Turnbull et al. (1998)

Trute & Hiebert‐Murphy (2002)

Walsh (2012)

Zhang, Yan, Barriball, While, & Liu, (2013)

**Positive aspects not described explicitly by parents (i.e. Hypotheses testing, relationships, correlations, models) (N=16)**

Blacher & Baker, (2007)

Blacher, Begum, Marcoulides, & Baker, 2013)

Byra, Zyta, & Cwirynkalo, (2017)

Ferrer, Vilaseca, & Bersabé, (2016)

Flaherty & Glidden, (2000)

Greenberg, Seltzer, & Greenley, (1993)

Grey, Totsika, & Hastings, (2017)

Kurtz-Nelson & McIntyre, (2017)

Friedrich & Friedrich, (1981)

Mak & Ho, (2007)

MacDonald, Hastings, & Fitzsimons, (2010)

Rajan & John, (2017)

Ricci & Hodapp, (2003)

Simmerman, Blacher, & Baker, (2001)

Sandler & Mistretta, (1998)

Van de Veek, Kraaij, & Garnefski, (2009)

**Positive aspects not main focus/Publication not relevant (n=24)**

Abbott & Meredith, (1986)

(Barnett, Clements, Kaplan-estrin, & Fialka, 2003)

Bayat, (2007)

Beresford, Rabiee, & Sloper, (2007)

Blacher & McIntyre, (2006)

Byrne & Cunningham, (1985)

Cairns et al., (2014)

Carr, 1988, 2008)

Dykens, (2005, 2006)

Gath, (1977)

Haring, Lovett, & Saren, (1991)

Heiman (2002)

Heller, Miller, & Factor, (1997)

Hodapp et al., (2001)

James (2013)

King & Patterson, (2000)

Knox & Parmenter, (2000)

McDermott et al., (1996)

McDermott, Valentine, Anderson, Gallup, & Thompson, (1997)

Seltzer, Krauss, & Taunematsu, (1993)

Pelchat et al., (2003)

Yannamani, Zia, & Khalil (2009)

**Unable to obtain full text (n=6)**

Blackard & Barsh, (1982)

Counselman-Carpenter, (2017)

Falik, (1995)

Lauderdale-Littin & Blacher, (2017)

McDermott, Valentine, Anderson, & Thompson, (1996)

McLinden, (1990)

**References:**

Abbott, D., & Meredith, W. (1986). Strengths of parents with retarded children. *Family Relations*, *35*, 371–375.

Barnett, D., Clements, M., Kaplan-estrin, M., & Fialka, J. (2003). Building New Dreams Supporting Parents’ Adaptation to Their Child With Special Needs. *Infants and Young Children*, *16*(3), 184–200. https://doi.org/10.1097/00001163-200307000-00002

Bayat, M. (2007). Evidence of resilience in families of children with autism. *Journal of Intellectual Disability Research*, *51*(9), 702–714. https://doi.org/10.1111/j.1365-2788.2007.00960.x

Behr, S. K., & Murphy, D. L. (1993). Research progress and promise: The role of perceptions in cognitive adaptation to disability. In *Cognitive coping, families, and disability* (pp. 151–164). https://doi.org/10.1172/JCI46043.rons

Bekhet, A. K., Johnson, N. L., & Zauszniewski, J. A. (2012). Effects on Resilience of Caregivers of Persons With Autism Spectrum Disorder: The Role of Positive Cognitions. *Journal of the American Psychiatric Nurses Association*, *18*(6), 337–344. https://doi.org/10.1177/1078390312467056

Beresford, B., Rabiee, P., & Sloper, P. (2007). *Outcomes for parents with disabled children*. York.

Blacher, J., & Baker, B. . (2007). Positive impact of intellectual disability on families. *Journal on Mental Retardation*, (112), 330–338.

Blacher, J., Begum, G. F., Marcoulides, G. A., & Baker, B. L. (2013). Longitudinal Perspectives of Child Positive Impact on Families: Relationship to Disability and Culture. *American Journal on Intellectual and Developmental Disabilities*, *118*(2), 141–155. https://doi.org/10.1352/1944-7558-118.2.141

Blacher, J., & McIntyre, L. L. (2006). Syndrome specificity and behavioural disorders in young adults with intellectual disability: cultural differences in family impact. *Journal of Intellectual Disability Research*, *50*(3), 184–198. https://doi.org/10.1111/j.1365-2788.2005.00768.x

Blackard, M., & Barsh, E. (1982). Parents and professional’s perceptions of the handicapped child’s impact on the family. *Journal of the Association for the Severely Handicapped*, *7*, 62–70.

Boström, P. K., Broberg, M., & Bodin, L. (2011). Child’s positive and negative impacts on parents—A person-oriented approach to understanding temperament in preschool children with intellectual disabilities. *Research in Developmental Disabilities*, *32*(5), 1860–1871. https://doi.org/10.1016/j.ridd.2011.03.017

Burton-Smith, R., Burton‐Smith, R., & McVilly, K. (2009). Quality of life of Australian family carers: implications for research, policy and practice. *Journal of Policy and Practice in Intellectual Disabilities*, *6*(3), 189–198. https://doi.org/10.1111/j.1741-1130.2009.00227.x

Byra, S., Zyta, A., & Cwirynkalo, K. (2017). Posttraumatic Growth in Mothers of children with disabilities. *Hrvatska Revija Za Rehabilitacijska Istraživanja*, *53*, 15–17.

Byrne, E., & Cunningham, C. (1985). The effects of mentally handicapped children on families: A conceptual review. *Journal of Child Psychology & Psychiatry*, *26*(6), 847–864.

Cairns, D., Brown, J., Tolson, D., & Darbyshire, C. (2014). Caring for a child with learning disabilities over a prolonged period of time: An exploratory survey on the experiences and health of older parent carers living in scotland. *Journal of Applied Research in Intellectual Disabilities*, *27*(5), 471–480. https://doi.org/10.1111/jar.12071

Carr, J. (1988). Six weeks to twenty-one years old: A longitudinal study of children with Down’s syndrome and their families. *Journal of Child Psychology and Psychiatry*, *29*(4), 407–431. https://doi.org/10.1111/j.1469-7610.1988.tb00734.x

Carr, J. (2008). Families of 40-Year Olds With Down Syndrome. *Journal on Developmental Disabilities*, *14*(2), 35–43.

Counselman-Carpenter, E. A. (2017). The presence of posttraumatic growth (PTG) in mothers whose children are born unexpectedly with Down syndrome. *Journal of Intellectual & Developmental Disability*, *42*(4), 351–363. https://doi.org/10.3109/13668250.2016.1247207

Davis, C. G., Nolen-Hoeksema, S., & Larson, J. (1998). Making sense of loss and benefiting from the experience: Two construals of meaning. *Journal of Personality and Social Psychology*, *75*(2), 561–574. https://doi.org/10.1037/0022-3514.75.2.561

Dillenburger, K., & McKerr, L. (2011). “How long are we able to go on?” Issues faced by older family caregivers of adults with disabilities. *British Journal of Learning Disabilities*, *39*(1), 29–38. https://doi.org/10.1111/j.1468-3156.2010.00613.x

Dykens, E. (2005). Happiness, Well-Being, and Character Strengths: Outcomes for Families and Siblings of Persons With Mental Retardation. *Mental Retardation*, *43*(5), 360–364. https://doi.org/10.1352/0047-6765(2005)43[360:HWACSO]2.0.CO;2

Dykens, E. M. (2006). Toward a positive psychology of mental retardation. *American Journal of Orthopsychiatry*, *76*(2), 185–193. https://doi.org/10.1037/0002-9432.76.2.185

Falik, L. (1995). Family patterns of reaction to a child with a learning disability: a mediational perspective. *Journal of Learning Disabilities*, *28*(6), 335–341.

Ferrer, F., Vilaseca, R., & Bersabé, R. M. (2016). The Impact of Demographic Characteristics and the Positive Perceptions of Parents on Quality of Life in Families with a Member with Intellectual Disability. *Journal of Developmental and Physical Disabilities*, *28*(6), 871–888. https://doi.org/10.1007/s10882-016-9515-z

Findler, L. (2014). The Experience of Stress and Personal Growth Among Grandparents of Children With and Without Intellectual Disability. *Intellectual and Developmental Disabilities*, *52*(1), 32–48. https://doi.org/10.1352/1934-9556-52.1.32

Flaherty, E., & Glidden, L. (2000). Positive adjustment in parents rearing children with Down Syndrome. *Early Education & Development*, *11*(4), 483–498. https://doi.org/10.1207/s15566935eed1104

Friedrich, W. N., & Friedrich, L. (1981). Psychosocial Assets of Parents of Handicapped Children. *American Journal of Mental Deficiency*, *85*(5), 551–553. https://doi.org/10.1037/0022-006X.47.6.1140

Gath, A. (1977). The impact of an abnormal child upon the parents. *The British Journal of Psychiatry*, *130*(4), 405–410. https://doi.org/10.1192/bjp.130.4.405

Glidden, L. M., & Natcher, A. L. (2009). Coping strategy use, personality, and adjustment of parents rearing children with developmental disabilities. *Journal of Intellectual Disability Research*, *53*(12), 998–1013. https://doi.org/10.1111/j.1365-2788.2009.01217.x

Green, S. E. (2007). “We’re tired, not sad”: Benefits and burdens of mothering a child with a disability. *Social Science and Medicine*, *64*(1), 150–163. https://doi.org/10.1016/j.socscimed.2006.08.025

Greenberg, J., Seltzer, M., & Greenley, J. (1993). Aging parents of adults with disabilities: The gratifications and frustrations of later-life caregiving. *Gerontologist*, *33*(4), 542–550.

Grey, J. M., Totsika, V., & Hastings, R. P. (2017). Physical and psychological health of family carers co-residing with an adult relative with an intellectual disability. *Journal of Applied Research in Intellectual Disabilities*, *34*(2), 299–307. https://doi.org/10.1111/jar.12353

Gupta, A., & Singhal, N. (2004). Positive perceptions in parents of children with disabilities. *Asia Pacific Disability Rehabilitation Journal*, *15*(1), 22–35.

Haring, K. A., Lovett, D. L., & Saren, D. (1991). Parent Perceptions of Their Adult Offspring with Disabilities. *Teaching Exceptional Children*, *23*(2), 6–10. https://doi.org/10.1177/004005999102300203

Hastings, R. P., Kovshoff, H., Ward, N. J., Espinosa, F. degli, Brown, T., & Remington, B. (2005). Systems Analysis of Stress and Positive Perceptions in Mothers and Fathers of Pre-School Children with Autism. *Journal of Autism and Developmental Disorders*, *35*(5), 635–644. https://doi.org/10.1007/s10803-005-0007-8

Hastings, R., & Taunt, H. (2002). Positive perceptions in families of children with developmental disabilities. *American Journal on Mental Retardation*, *107*(2), 116–127.

Heiman, T. (2002). Parents of children with disabilities: Resilience, coping and future expectations. *Journal of Developmental and Physical Disabilities*, *14*(2), 159–171. https://doi.org/10.1023/A:1015219514621

Heller, T., Miller, A., & Factor, A. (1997). Adults with mental retardation as supports to their parents: effects on parental caregiving appraisal. *Mental Retardation*, *35*(5), 338.

Hodnapp, R. ., Ly, T. . M., Fidler, D. . J., Ricci, L. . A., Hodapp, R. M., Ly, T. . M., … Ricci, L. . A. (2001). Less stress, more rewarding: Parenting children with Down syndrome. *Parenting Science and Practice*, *1*(4), 317–337.

James, N. (2013). How families perceive the care-giving experience. *Learning Disability Practice*, *16*(3), 32–37. https://doi.org/10.7748/ldp2013.04.16.3.32.e1416

Kayfitz, A. D., Gragg, M. M. N., Orr, R., & Robert Orr, R. (2010). Positive Experiences of Mothers and Fathers of Children with Autism. *Journal of Applied Research in Intellectual Disabilities*, *23*(4), 337–343. https://doi.org/10.1111/j.1468-3148.2009.00539.x

King, G., Zwaigenbaum, L., Bates, a., Baxter, D., & Rosenbaum, P. (2012). Parent views of the positive contributions of elementary and high school-aged children with autism spectrum disorders and Down syndrome. *Child: Care, Health and Development*, *38*(6), 817–828. https://doi.org/10.1111/j.1365-2214.2011.01312.x

King, L. A., & Patterson, C. (2000). Reconstructing life goals after the birth of a child with Down’s syndrome: finding happiness and growing. *International Journal of Rehabilitation and Health*, *5*(1), 17–30.

Knox, M., & Parmenter, T. (2000). Family control: The views of families who have a child with an intellectual disability. *Journal of Applied Research in Intellectual Disabilities*, (13), 17–28.

Konrad, S. C. (2006). Posttraumatic Growth in Mothers of Children with Acquired Disabilities. *Journal of Loss and Trauma*, *11*(1), 101–113. https://doi.org/10.1080/15325020500358274

Krauss, M. W., Seltzer, M. M., & Jacobson, H. T. (2005). Adults with autism living at home or in non-family settings: positive and negative aspects of residential status. *Journal of Intellectual Disability Research*, *49*(2), 111–124. https://doi.org/10.1111/j.1365-2788.2004.00599.x

Kurtz-Nelson, E., & McIntyre, L. L. (2017). Optimism and positive and negative feelings in parents of young children with developmental delay. *Journal of Intellectual Disability Research*, *61*(7), 719–725. https://doi.org/10.1111/jir.12378

Lauderdale-Littin, S., & Blacher, J. (2017). Young adults with severe intellectual disability: Culture, parent, and sibling impact. *Journal of Intellectual & Developmental Disability*, *42*(3), 230–239. https://doi.org/10.3109/13668250.2016.1230843

MacDonald, E. E., Hastings, R. P., & Fitzsimons, E. (2010). Psychological acceptance mediates the impact of the behaviour problems of children with intellectual disability on fathers’ psychological adjustment. *Journal of Applied Research in Intellectual Disabilities*, *23*(1), 27–37. https://doi.org/10.1111/j.1468-3148.2009.00546.x

Mak, W. W. S., & Ho, G. S. M. (2007). Caregiving Perceptions of Chinese Mothers of Children with Intellectual Disability in Hong Kong. *Journal of Applied Research in Intellectual Disabilities*, *20*(2), 145–156. https://doi.org/10.1111/j.1468-3148.2006.00309.x

Manor-Binyamini, I. (2014). Positive aspects of the coping of mothers of adolescent children with developmental disability in the Bedouin community in Israel. *Research in Developmental Disabilities*, *35*(6), 1272–1280. https://doi.org/10.1016/j.ridd.2014.03.018

Manor-Binyamini, I. (2016). Positive aspects of coping among mothers of adolescent children with developmental disability in the Druze community in Israel. *Journal of Intellectual and Developmental Disability*, *41*(2), 97–106. https://doi.org/10.3109/13668250.2015.1129665

McConnell, D., Savage, A., Sobsey, D., & Uditsky, B. (2015). Benefit-finding or finding benefits? The positive impact of having a disabled child. *Disability & Society*, *30*(1), 29–45. https://doi.org/10.1080/09687599.2014.984803

McDermott, S., Territo, T., Valentine, D., Anderson, D., Gallup, D., & Thompson, S. (1996). Aging parents of adult children with mental retardation:Is age a factor in their perception of burdens and gratifications? *Journal of Gerontological Social Work*, *27*((1/2)), 133–148.

McDermott, S., Valentine, D., Anderson, D., Gallup, D., & Thompson, S. (1997). Parents of adults with mental retardation living in home and out of home:Caregiver burdens and gratifications. *American Journal of Orthopsychiatry*, *67*(2), 323–329.

McDermott, S., Valentine, D., Anderson, D., & Thompson, S. (1996). Does residential placement of adult children with mental retardation influence the burden and gratifications of their mothers? *Adult Residential Care Journal*, *10*(2), 102–114.

McLinden, S. (1990). Mothers’ and fathers’ reports of the effects of a young child with special needs on the family. *Journal of Early Intervention*, *14*(3), 249–259. https://doi.org/https://doi.org/10.1177/105381519001400306

Mullins, J. (1987). Authentic voices from Parents of exceptional children. *Family Relations*, *36*, 30–33.

Neely-Barnes, S. ., & Dia, D. . (2008). Families of children with disabilities: A review of literature and recommendations for interventions. *Journal of Early and Intensive Behavior Intervention*, *5*(3), 93–107. https://doi.org/10.1037/h0100425

Nelson, A. M. (2002). A Metasynthesis: Mothering Other-than-Normal Children. *Qualitative Health Research*, *12*(4), 515–530. https://doi.org/10.1177/104973202129120043

Pelchat, D., Lefebvre, H., & Perreault, M. (2003). Differences and similarities between mothers’ and fathers’ experiences of parenting a child with a disability. *Journal of Child Health Care*, *7*(4), 231–247. https://doi.org/10.1177/13674935030074001

Phelps, K. W., McCammon, S. L., Wuensch, K. L., & Golden, J. A. (2009). Enrichment, stress, and growth from parenting an individual with an autism spectrum disorder*. *Journal of Intellectual and Developmental Disability*, *34*(2), 133–141. https://doi.org/10.1080/13668250902845236

Picoraro, J. A., Womer, J. W., Kazak, A. E., & Feudtner, C. (2014). Posttraumatic Growth in Parents and Pediatric Patients. *Journal of Palliative Medicine*, *17*(2), 209–218. https://doi.org/10.1089/jpm.2013.0280

Poehlmann, J., Clements, M., Abbeduto, L., & Farsad, V. (2005). Family Experiences Associated With a Child’s Diagnosis of Fragile X or Down Syndrome: Evidence for Disruption and Resilience. *Mental Retardation*, *43*(4), 255–267. https://doi.org/10.1352/0047-6765(2005)43[255:FEAWAC]2.0.CO;2

Rajan, A. M., & John, R. (2017). Resilience and impact of children’s intellectual disability on Indian parents. *Journal of Intellectual Disabilities*, *21*(4), 315–324. https://doi.org/10.1177/1744629516654588

Ricci, L. A., & Hodapp, R. M. (2003). Fathers of children with Down’s syndrome versus other types of intellectual disability: Perceptions, stress and involvement. *Journal of Intellectual Disability Research*, *47*(4–5), 273–284. https://doi.org/10.1046/j.1365-2788.2003.00489.x

Sandler, A., & Mistretta, L. (1998). Positive adaptation in parents of adults with disabilities. *Education and Training in Mental Retardation and Developmental Disabilities*, *33*(2), 123–130.

Sarriá, E., & Pozo, P. (2015). Coping Strategies and Parents’ Positive Perceptions of Raising a Child with Autism Spectrum Disorders. In M. Fitzgeral (Ed.), *Autism Spectrum Disorder - Recent Advances*. https://doi.org/10.5772/58966

Scorgie, K., & Sobsey, D. (2000). Transformational outcomes associated with parenting children who have disabilities. *Mental Retardation*, *38*(3), 195–206.

Scorgie, K., & Wilgosh, L. (2008). Reflections on an uncommon journey: A follow up study of life management of six mothers of children with diverse disabilities. *International Journal of Special Education*, *23*(1), 103–114.

Seltzer, M., Krauss, M., & Taunematsu, N. (1993). Adults with Down syndrome and their aging mothers: Diagnostic group differences. *American Journal on Mental Retardation*, *97*(5), 496–508.

Simmerman, S., Blacher, J., & Baker, B. (2001). Fathers’ and mothers’ perceptions of father involvment in families with young children with a disability. *Journal of Intellectual and Developmental Disability*, *26*(4), 325–338. https://doi.org/10.1080/1366825012008733

Slattery, É., McMahon, J., & Gallagher, S. (2017). Optimism and benefit finding in parents of children with developmental disabilities: The role of positive reappraisal and social support. *Research in Developmental Disabilities*, *65*(April), 12–22. https://doi.org/10.1016/j.ridd.2017.04.006

Strecker, S., Hazelwood, Z. J., & Shakespeare-Finch, J. (2014). Postdiagnosis personal growth in an Australian population of parents raising children with developmental disability. *Journal of Intellectual and Developmental Disability*, *39*(1), 1–9. https://doi.org/10.3109/13668250.2013.835035

Trute, B., & Hiebert‐Murphy, D. (2002). Family adjustment to childhood developmental disability: A measure of parental appraisal of family impacts. *Journal of Pediatric Psychology*, *27*(3), 271–280.

Trute, B., Hiebert‐Murphy, D., & Levine, K. (2007). Parental appraisal of the family impact of childhood developmental disability: Times of sadness and times of joy. *Journal of Intellectual and Developmental Disability*, *32*(1), 1–9. https://doi.org/10.1080/13668250601146753

Turnbull, H. ., Guess, D., & Turnbull, A. P. (1988). Vox Populi and Baby Doe. *Mental Retatdation*, *26*(3), 127–132.

Van de Veek, S. M., Kraaij, V., & Garnefski, N. (2009). Down or up ? Explaining positive and negative emotions in parents of children with Down ’ s syndrome : Goals , cognitive coping , and resources. *Journal of Intellectual & Developmental Disability*, *34*(September), 216–229. https://doi.org/10.1080/13668250903093133

Walsh, F. (2012). Family Resilience: Strengths through adversity. In F. Walsh (Ed.), *Normal Family Processess* (pp. 399–427). New York: Guildford Press.

Yannamani, N., Zia, A., & Khalil, N. (2009). Family carers of people with learning disabilities: Common themes across caring. *Psychiatry*, *8*(11), 441–444. https://doi.org/10.1016/j.mppsy.2009.07.007

Zhang, W., Yan, T. T., Barriball, K. L., While, A. E., & Liu, X. H. (2013). Post-traumatic growth in mothers of children with autism: A phenomenological study. *Autism : The International Journal of Research and Practice*, (November). https://doi.org/10.1177/1362361313509732
